# Investigating *hsp* Gene Expression in Liver of *Channa striatus* under Heat Stress for Understanding the Upper Thermal Acclimation

**DOI:** 10.1155/2014/381719

**Published:** 2014-06-09

**Authors:** Gopal Krishna Purohit, Arabinda Mahanty, Mrutyunjay Suar, Anil Prakash Sharma, Bimal Prasanna Mohanty, Sasmita Mohanty

**Affiliations:** ^1^School of Biotechnology, KIIT University, Bhubaneswar, Odisha 751024, India; ^2^Biochemistry Laboratory, ICAR-Central Inland Fisheries Research Institute, Barrackpore, Kolkata 700120, India

## Abstract

Changes in *hsp* gene expression profiles in murrel *Channa striatus* experimentally exposed to temperature stress (36°C) for 4, 15, and 30 days were investigated; fish collected from aquaculture ponds and maintained in laboratory at the pond temperature (25 ± 1°C) served as control. *Channa* collected from a hot spring runoff (36°C) was included in the study to examine the *hsp* profiles beyond 30 days of exposure. Gene expression analyses of a battery of *hsps* in liver tissues were carried out by quantitative RT-PCR and protein expressions were analyzed by immunoblotting. *hsps* could be grouped into three clusters based on similarity in response to heat stress: *hsp70, hsp78, * and * hsp60*, whose transcript level continued to increase with duration of exposure; *hsp90* and *hsp110* that increased to a much higher level and then decreased; *hsp27* and *hsp47* that did not significantly vary as compared to control. The results suggest that Hsp70, Hsp78, and Hsp60 are involved in thermal acclimation and long term survival at high temperature. Fish living in the hot spring runoff appears to continuously express *hsps* that can be approximated by long term induction of *hsps* in farmed fish if temperature of their environment is raised to 36°C.

## 1. Introduction

Climate change and resulting global warming are the most challenging environmental problems of the twenty-first century [1, 2]. Understanding the survival mechanisms and developing mitigation strategies are necessary, as, if an organism has to survive, it must be able to adapt to the changes in its environment. One possible cellular mechanism that could influence the upper thermal tolerance of an organism is the heat shock response (HSR) [3]. The HSR is a highly conserved cellular response in which the transcription and translation of heat shock proteins (Hsps) are induced by a variety of protein-denaturing stressors [3]. Heat shock proteins are involved in a variety of stress related processes, including withstanding thermal stress, scavenging oxygen radicals, induction of apoptosis, and mounting of immune response [3, 4]. In the unstressed cell, these proteins have constitutive functions that are essential in various aspects of protein metabolism and they also play important role in normal growth and development. In contrast to HSR, which causes transient enhancement of Hsps in response to sudden heat shock, acclimation response refers to physiological changes that happen when the organism is subjected to temperature fluctuations over weeks or months [5].

In natural ecosystems like the hot spring runoffs, the heat stress is almost perennial as the stressor is present almost round the year. We located one such hot spring, the Atri hot spring (20°09′N 85°18E), in the Khurda district of Odisha, India, during a survey, [6, 7] where the hot spring, which drains to a nearby rivulet, remains at a temperature of 36–38°C. Experimental fishing showed the presence of fishes like the murrel* Channa striatus* and minnow* Puntius sophore,* amongst others, as the predominant ones [7]. The fishes here face a temperature of about 36°C whereas the temperature in tropical ponds remains at 25–27°C round the year, barring the peak summer. Since these fishes feed, breed, and grow in this heat-stressed environment, they appear to have adapted to the stressed ecosystem. Understanding the thermotolerance mechanism in these lower vertebrates could add to our understanding of upper thermal acclimation and might be useful for devising methods to cope with global warming and heat stroke syndromes which have become public health problems these days [8–10].

In this backdrop, we investigated the* hsp* gene expression profiles in the murrel* Channa striatus* collected from the hot spring runoff water (36°C temperature) and the same fish collected from aquaculture ponds (25–27°C) and maintained in the laboratory at 25°C; both groups served as nonheat shocked controls. The* hsp* expression profiles of these fishes were compared to* Channa* collected from local farms and exposed to 36°C under laboratory conditions for various time periods, namely, 4, 15, and 30 days (heat shocked). We specifically analysed the* hsp* genes with the objective of identifying the Hsps, which could be the possible key players in the upper thermal acclimation and acquired thermotolerance. As* Channa striatus* was found to be available in most of the experimental fishing, suggesting that this species has possibly acclimatized well to the hyper thermal spring environment, we selected this species for the study. We attempted to address few questions like: how the* hsps* in* Channa* respond to high temperature stress on a short term basis? How the* hsp* expressions are modulated if the fish is exposed to high temperature for longer periods? Do the* hsps* behave in a similar manner when the temperature remains perennially high?

## 2. Experimental Procedures

### 2.1. Experimental Animals

Apparently healthy* Channa striata *(length 9.43 ± 1.43 cm, weight 50.25 ± 3.20 g) were collected from a local farm and were acclimatized in the laboratory for 15 days prior to the thermal exposure. During the acclimatization, holding conditions of 25 ± 1°C and 12 h : 12 h (light : dark cycle) was maintained and fishes were fed with a laboratory prepared feed containing crude protein (40%) and crude lipid (15%) at 3% of live weight and live* tubifex*. The study including sample collection, animal experimentation, and sacrifice was approved by the Animal Ethics Committee of the KIIT University. Liver tissues were collected from* Channa striatus* collected from the hot spring runoff and control after euthanizing them with MS-222 (200 mg/L), flash frozen in liquid nitrogen, and stored at −80°C for further experimentation.

### 2.2. Thermal Exposure

The fishes were randomly divided into four experimental groups, each group having nine fishes. The experimental exposures were carried out in metal aquarium (dimension: water capacity 75 L) with 30 L fresh water for duration of exposure. Control group had the normal holding temperature of 25 ± 1°C while the other groups were maintained at 36°C for 4, 15, and 30 days, respectively. Each day, one-third of the aged water was renewed with fresh water of 36°C or 25°C (for control). The tanks were fitted with aerators to avoid stress due to low oxygen tension. No mortality was observed in any treatment group during the exposure period. Fishes were sampled from different groups (4/15/30 days) at the end of exposure period, euthanized with MS-222 (200 mg/L) and liver tissues were dissected out. The tissues were immediately stored at –40°C for immunoblotting or flash frozen in liquid nitrogen for RNA extraction followed by storage at –80°C.

### 2.3. Total RNA Extraction & Semiquantitative Reverse Transcriptase PCR Amplification

Total RNA was extracted from liver using the RiboZol (Himedia Laboratories, India) following manufacturer's protocol. Following isolation of RNA, the concentration of each RNA sample was measured by Nanodrop (Biotech instruments, USA) and the quality was accessed by electrophoresis in an agarose formaldehyde gel (1% w/v agarose, 16% formaldehyde). RNA was stored at −80°C for further use. RNA samples were treated with the DNase 1 (NEB, UK) as per the manufacturer's recommended protocol to remove potential genomic DNA carryover.

Primers used for the amplification of target cDNAs were designed from conserved regions of related sequences available in the databank (http://www.ncbi.nlm.nih.gov/). GenBank information used for designing the* Channa hsps* are as stated:* hsp70* -* Danio rerio:* AB062116,* Lates calcarifer:* HQ646109,* Sciaenops ocellatus:* GU244375,* Oryzias latipes*: AF286875,* Cyprinus carpio*: AY120894, and* Salmo salar*: AJ632154;* hsp90* -* Danio rerio*: L35586.1,* Cyprinus carpio*: AF170295,* Carassius carassius*: FM995214, and* Ctenopharyngodon idella*: FJ517554;* hsp78* -* Rattus norvegicus*: M14866,* Mus musculus*: U16277,* Homo sapiens*: BC112963,* Ovis aries*: XM_004005637;* hsp110 - Sus scrofa*: NM_001097504,* Cavia porcellus*: XM_003477263,* Ornithorhynchus anatinus*: XM_001509005, and* Pan troglodytes*: XM_003314105;* hsp27* -* Carassius auratus*: DQ872651.1,* Danio rerio*: NM_001008615, and* Kryptolebias marmoratus*: HQ213889. Primer pairs for* hsp60* and* hsp47* were taken from Healy et al. [11] and Wang et al. [12], respectively. All sequences were aligned with ClustalW using Bio edit sequence alignment editor 7.0.5.3 software and primers were designed with the help of Primer3 software (http://frodo.wi.mit.edu/). The primer pair that produced a single and prominent amplification product was taken for further analysis.

RNA (1 *μ*g) was reverse transcribed using M-MLV reverse transcriptase (Promega, USA) according to the manufacturer's protocol. PCR in a 50 *μ*L mixture consisted of 20 ng of first strand cDNA, 1X hot start buffer (200 mM Tris-HCl (pH 8.3), 500 mM MgCl_2_ (pH 8.5)), 200 *μ*M each of dNTPs, 10 pM of each gene specific primer, and 5 U of hot start polymerase. PCR analysis was carried out by using a gradient thermal cycler (Eppendorf, Germany). The amplification conditions were as follows: 5 min of predenaturation at 95°C followed by 40 cycles of amplification (denaturation at 95°C for 20 s, annealing for 20 s at temperatures optimized for specific genes (Table 1), and extension at 72°C for 1 min) and a final extension at 72°C for 1 min.

### 2.4. Sequencing of* hsps*

PCR products were electrophoresed on 1.5% agarose gels and bands of appropriate size were purified from the gels using gel extraction kit (Himedia Laboratories, India). They were further sequenced by using Sanger's dideoxy sequencing protocol (ABI 3730 XL) with specific primers. Partial sequences of the respective genes were submitted to GenBank and the consensus sequences were used for further analysis. To confirm the identity of the consensus* hsps*, the sequences were subjected to Blastn and Blastx [13] for comparing with the GenBank nucleotide and protein database.

### 2.5. Quantitative Real Time PCR Analysis of* hsp* Gene Expression

For the gene expression analysis by qPCR, primers for* hsp70*,* hsp90*,* hsp60, hsp78*,* hsp110, hsp47,* and* hsp27 *were redesigned from partial sequences of the respective genes deposited in GenBank. All qRT-PCR primers were designed using Primer3 software and are given in Table 1.

The reactions were carried out in CFX connect real time PCR detection system (Bio-Rad, UK). Each 20 *μ*L reactions contained 1X iQsyber green super mix (Bio-Rad, UK), 1 *μ*L (0.2 *μ*m) of each forward and reverse primer (Table 1), 2 *μ*L (200 ng) cDNA, and molecular grade water. All reactions were carried out according to the supplier's instructions. The housekeeping gene* tubulin* was used as an internal control for all real time PCR experiments (Table 1). The cycling parameters used were as follows: an initial denaturation (3 min at 95°C), followed by 40 cycles of 10 s at 95°C, 30 s at optimized temperatures for specific genes, and 30 s at 72°C with fluorescence measured at the end of the annealing and extension steps. At the end of each qRT-PCR reaction, PCR products were subjected to a melt curve analysis to confirm the presence of a single amplicon. Melting curve analysis was performed by increasing the temperature from 65°C to 95°C at a rate of 0.2°C/sec with continuous fluorescence at every 0.5°C increments. All samples were run in triplicate. The basal expression of each* hsp* (control) was taken to be unit and the relative-fold expression of* hsps* of heat shocked fish were calculated using the relative expression software (CFX Manager Software, Bio-Rad, UK). The comparative Cq (ΔCq) method was used to calculate the changes in gene expression as a relative-fold difference between the control and treated sample. All the procedures of real time PCR were performed following MIQE guidelines [14].

### 2.6. Protein Extraction, SDS-PAGE, and Immunoblot Analysis

Frozen fish liver was homogenized in ice-cold PBS (137 mM NaCl, 2.7 mM KCl, 4.3 mM Na_2_HPO_4_·7H_2_O, and 1.4 mM KH_2_PO_4_), pH 7.3 containing protease inhibitor cocktail (Sigma P8340) [15]. The homogenates were centrifuged at 11500 × g at 4°C for 15 min in high-speed refrigerated centrifuge. The supernatant was collected and stored at –20°C. To minimize protein modification or degradation, all dissection and sample processing was performed on ice. The protein content of each supernatant (soluble protein fraction) was determined by Bradford method [16], using BSA as the standard.

The soluble liver proteins were separated by SDS-PAGE [17] (12% w/v polyacrylamide gel with a 5% w/v stacking gel) at a constant potential of 200 V on a mini-Protean 3-electrophoresis cell (Bio-Rad). Protein Molecular weight markers (58445, Sigma) were co-run with the samples. Gels were stained with Coomassie Brilliant Blue R 250 (CBB) for visualization of the protein bands.

Immunoblot analyses were carried out to identify the changes in heat shock proteins (Hsps) expressed in the liver [18]. Transfer of proteins was carried out on an iBlot (Invitrogen) transfer apparatus using the kits and protocols of the manufacturer. Transfer was confirmed by staining the NC membrane with Ponceau S (P7170, Sigma). Immunoblotting was carried out on a Snap ID (Merck-Millipore) following the manufacturer's protocol. Monoclonal anti-Hsp90, -Hsp70, and -Hsp60 (H1775, H5147, H4149, Sigma) were used as the primary antibodies; anti-mouse IgG-peroxidase conjugate (A2304, Sigma) was used as the secondary antibody for Hsp60, Hsp70, and Hsp90. For *β*-actin which is a house keeping protein (control) monoclonal anti-*β*-actin (A3854) and anti-mouse IgG-peroxidase conjugate (A2304, Sigma) were used as primary and secondary antibodies, respectively. DAB (Sigma D-7304) and H_2_O_2_ were used as substrates.

After visualisation of the bands, the NC membranes were scanned using an image scanner (Image scanner III, GE Healthcare) and the relative-fold changes in the level of expression of the Hsps were analysed by using the software Image Quant TL 7.0.

### 2.7. Statistics

ΔCT values were used to determine the expression level of all* hsp* mRNAs from* Channa striatus* liver tissues. The variation among the different treatments such as the control, the treated, and of the Atri hot spring samples were analyzed by one-way analysis of variance (ANOVA) followed by Student's* t*-test using the Sigma plot statistical software (v. 10.X). All data are reported as mean ± SD and the level of statistical significance was set at *P* < 0.05.

## 3. Results 

### 3.1. Partial DNA Sequences of* Channa striatus hsps*

The partial sequencing of* hsp70, hsp90, hsp60*,* hsp78*,* hsp110*,* hsp47,* and* hsp27* genes was done and the sequence information has been deposited in GenBank (Table 2).

### 3.2. Effect of Heat Shock on* hsp70, hsp78*, and* hsp60* Gene Expression

**Figure 1:**
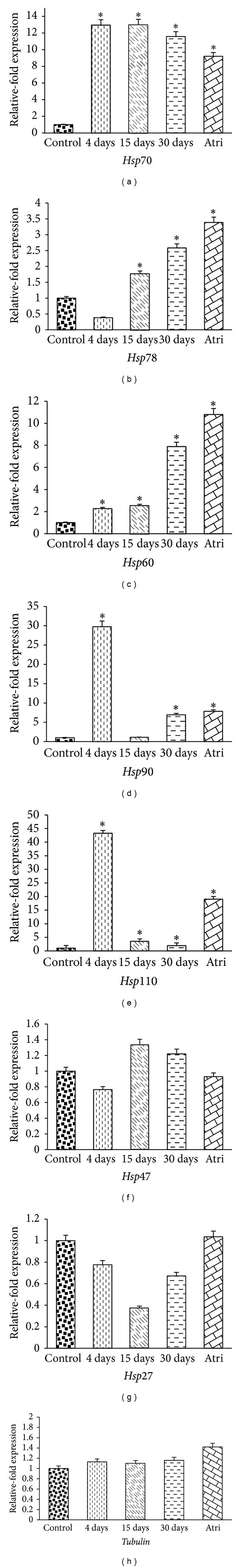
*hsp *gene expression analysis in* Channa striatus* experimentally exposed to high temperature (36°C) and those collected from hot spring runoff. Real time RT-PCR analysis of* hsp* transcripts in liver tissues of the heat-stressed fishes (36°C for 4-, 15-, and 30-days) and those collected from the Atri hot spring runoff was carried out (marked Atri); (a)* hsp70*, (b)* hsp78*, (c)* hsp60*, (d)* hsp90*, (e)* hsp110,* (f)* hsp47*, (g)* hsp27*, and (h)* tubulin* (control). All data were expressed as mean ± standard error (*n* = 9 for each experimentally exposed group and *n* = 6 for hot spring runoff collected fish) relative to control (*Channa* were collected from aquaculture ponds, water temperature 25°C and were maintained at 25°C in the laboratory), which was set as unit. Asterisks (*) denote statistically significant difference between control and experimentally exposed samples/fish collected from hot spring (*P* < 0.05).

**Figure 2:**
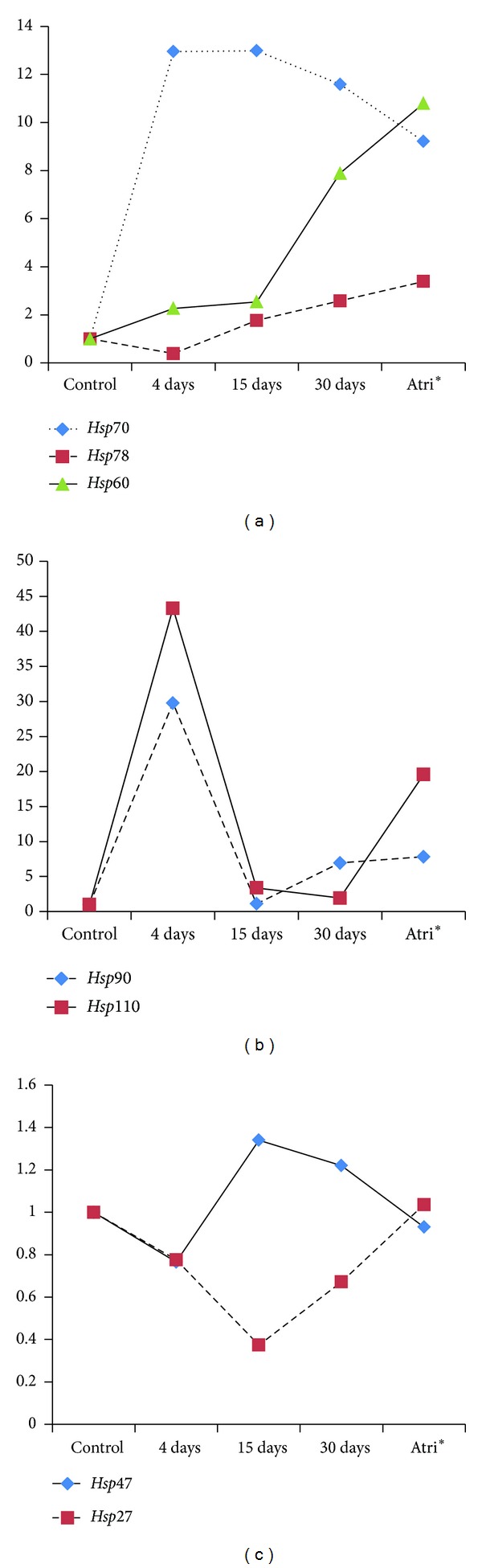
Trends in* hsp* gene expression in liver tissues of* Channa striatus* in response to heat stress. The* hsps* have been grouped into three clusters (a), (b), and (c) based on their similarity/near similarity in response to the heat stress; (a)* hsp70, hsp78,* and* hsp60*; (b)* hsp90* and* hsp110*; and (c)* hsp27* and* hsp47*. *Atri-fish collected from the Atri hot spring runoff.

Under the heat shock condition, the mRNA level of* hsp70* in liver tissue at 4 days exposure increased by 12.9-fold and remained similar at 15 days exposure as compared to control. When exposed for 30 days,* hsp70* expression level slightly decreased as compared to their levels at 4- and 15-day exposure; however, it was 10.59-fold higher as compared to control (Figure 1(a)). In hot spring runoff fishes,* hsp70* transcript level was at 9-fold (*P* = 0.0169) higher relative to control (Figures 1(a) and 2(a)).

The* hsp78* showed a decrease (0.3-fold) in its expression compared to control at 4 days of heat shock. As the exposure continued for longer periods, that is, 15 days and 30 days,* hsp78* gene expression increased by 1.5- and 2.5-fold, respectively (Figures 1(b) and 2(a)). The expression level in hot spring runoff fishes was 3.38-folds higher as compared to the 25°C control.

During 4 days and 15 days exposure of* Channa* to 36°C, there was gradual increase in* hsp60* gene expression with increase in duration of thermal exposure. It was 2.27-, 2.5-, and 7.8-folds (*P* = 0.0169) at 4-, 15- and 30-day exposure, respectively (Figure 1(c)). Further upregulation in* hsp60* expression (10.79-folds) was noted in hot spring runoff fish (Figures 1(c) and 2(a)).

### 3.3. Effect of Heat Shock on* hsp90* and* hsp110* Gene Expression

The* hsp90* gene expression was significantly up-regulated (29.7 fold increase relative to control; *P* ≤ 0.0001) when fish were exposed to heat stress for 4 days; however, its expression level decreased and became almost same as control at 15 days. The expression level increased by 6.9-folds (Figure 1(d)) at 30 days of exposure. In the hot spring runoff fish,* hsp90* level remained high (7.8-folds) as compared to control (Figures 1(d) and 2(b)). A Blastx search with our amplified partial* hsp90* sequence indicated that it was similar to Hsp90*α* protein.

The expression of* hsp110* transcript level was significantly up regulated (43-fold: *P* ≤ 0.0001) at 4 days of thermal exposure as compared to control; however, it decreased gradually with increase in duration of exposure, from 3.4-fold at 15 days to 1.9-fold at 30 days (Figure 1(e)). Surprisingly,* hsp110* transcript level in hot spring runoff fish showed 19-fold increase relative to control (Figures 1(e) and 2(b)).

### 3.4. Effect of Heat Shock on* hsp47* and* hsp27* Gene Expression

The* hsp47* transcript level decreased at 4 days of exposure as compared to control; it showed increase (1.4-fold) at 15 days of exposure and 1.2-fold at 30 days of exposure (Figures 1(f) and 2(c)). It was the same as control in the hot spring runoff fish. The* hsp27* transcript level decreased at 4 days and continued to decrease at 15 days of thermal exposure relative to control; however, The expression level slowly increased towards baseline level of expression as the exposure period was extended for 30 days (Figures 1(g) and 2(c)). In hot spring runoff fish, the level of* hsp47 *and* hsp27* transcripts were similar to control (Figure 1(g)).

### 3.5. Changes in Hsp70, Hsp90, and Hsp60 Protein Levels in* Channa* following Thermal Exposure

**Figure 3:**
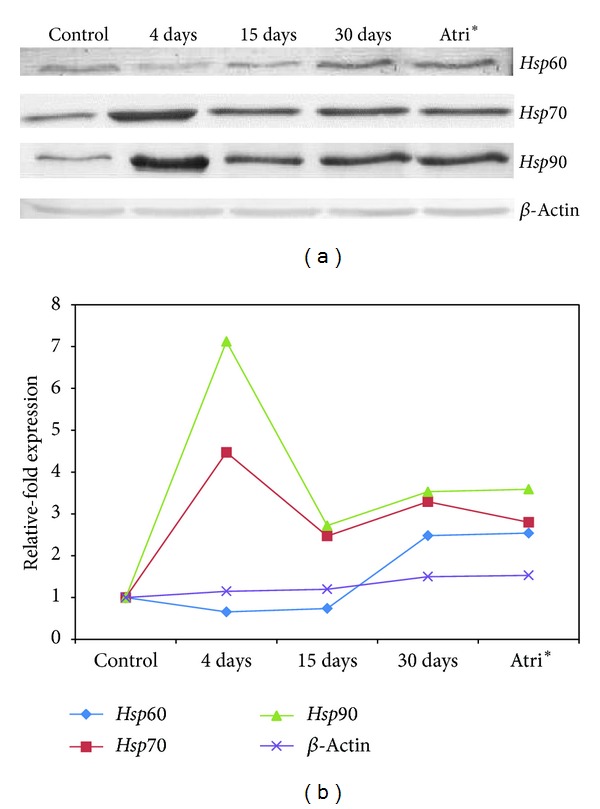
(a) Immunoblot analysis of Hsp60, Hsp70, Hsp90, and *β*-actin proteins in* Channa striatus* under heat stress. The variations in the protein levels in the fish exposed to high temperature (36°C) for 4, 15, and 30 days and those collected from the hot spring runoff (Atri) as compared to control (*Channa* collected from aquaculture ponds, 25°C) are shown. (b) Relative-fold change in the Hsp60, Hsp70, Hsp90, and *β*-actin (control) protein expression. *Atri-fish collected from the Atri hot spring runoff.

To examine the changes occurring at translational level, Hsp70, Hsp90 and Hsp60 proteins were analyzed by employing immunoblot analysis. Compared to 25°C control, Hsp70 and Hsp90 protein bands increased significantly in intensity in 4 days-exposed fish liver while the intensity of protein bands decreased at 15 and 30 days of thermal exposure and in hot spring runoff fish (Figure 3); however it was higher than the control fishes. Hsp60 protein expression was low in 4 and 15 days of heat shocked fish liver while the level was high in 30 days heat shocked and hot spring runoff fish (Figure 3).

## 4. Discussion

Fishes are excellent vertebrate models and have been extensively used for investigations of the effect of temperature fluctuations on the physiology and function as well as regulation of Hsps [19, 20]. One of the major types of cellular damage resulting from stress due to diverse abiotic factors, including temperature, is denaturation of proteins [21]. Consequently, adaptation to this stress leads to the elevated expression of heat shock genes that produce the heat shock proteins, which in turn interact with stress-denatured proteins to maintain or restore their native structures and prevent aggregation and degradation [21].

In the present study, we showed that in short term (4 days) as well as long term (15 and 30 days and beyond) acclimation to heat shock, modulation in expression of different* hsps* is taking place in liver tissue of* Channa striatus.* For the experiments, liver was chosen as it is a metabolically active tissue with respect to protein synthesis and turnover. Following warm temperature acclimation, significant up regulation of genes at their transcript and protein levels has been found to be taking place in liver tissue of fish in several studies [22].

In response to heat stress,* hsp70* transcript level was up regulated by 12.9-fold at 4 days of thermal exposure as compared to control, which was almost maintained when the exposure period was extended to 15 and 30 days. Several studies have shown the correlation of the* hsp70* gene expression with changing environments [23, 24]. It is the primary family of heat shock proteins that is induced by thermal stress [25, 26]. In our study, high level of* hsp70* transcript induction at 4, 15, and 30 days of thermal exposure is indicative of the protective role played by it in response to both short term and long term heat stress.

The results of transcript analysis were compared with Hsp70 protein expression by immunoblot analysis for validation. Compared to control, protein level of Hsp70 was significantly up regulated (4-fold) at 4 days of exposure; it was slowly down regulated; however it did not return to control level. The Hsp protein level was also found to be 2.5- to 3-fold higher than control, suggesting that Hsp70 is involved in short term and long term acclimation as well as survival [23] (Figures 3(a) and 3(b)). Changes in the rate of Hsp70 protein synthesis and catabolism may be important regulatory components of the long term modulation in Hsp70 level [23].

*hsp70* transcripts in the fishes from the hot spring showed similar levels of expression as that of 30 days of laboratory exposure indicating that it continues to be expressed at a higher basal level in the hot spring runoff environment. This appears to be an adaptive mechanism as similar observations have been reported in the Antarctic fishes also, where the induced form of* hsp70* is constitutively expressed [27].

The molecular chaperone Hsp78, which confers compartment-specific thermo tolerance to mitochondria, is crucial for maintenance of respiratory competence and for mitochondrial genome integrity under severe temperature stress [28]. The* hsp78* levels increased in a linear fashion and correlated with water temperatures in white muscle of rainbow trout (*Oncorhynchus mykiss*) [29] and in green sturgeon,* Acipenser medirostris* larvae, on continuous exposure to 26°C [30]. As shown in Figure 1, we got a consistent increase in expression of liver* hsp78* mRNA when* Channa* were continuously exposed to 36°C for 1 month. Interestingly, this high level of expression was observed in the fish collected from the hot spring runoff and* hsp78* transcript levels remained at level (3.5-fold) as compared to control indicating that this Hsp is an important player in thermal acclimation and adaptation process.

The* hsp60* transcript is up regulated in response to high temperature treatment in different organisms including fish [19, 31, 32]. In the present investigation, we observed a consistent upregulation of the* hsp60* transcript, the level being highest at 30 days of thermal exposure relative to control (Figures 1(c) and 2(a)). The protein level also correspondingly increased as the time of exposure was extended (Figure 3) which is in contrast with the study conducted by Logan and Somero [32] where* hsp60* expression was increased shortly after heat shock but decreased with time in fish* Gillichthys mirabilis*. In* Channa*, Hsp60 might have a greater role to play in the acclimation at higher temperatures and therefore it is continuously synthesized depending upon the need thereby aiding survival at a higher temperature. Like the* hsp78, hsp60* transcript levels were over expressed and showed a 10.79-fold increase in the liver of fishes collected from the hot spring runoff. Western blot analysis showed that the level of Hsp60 expression decreased in the fishes exposed to 36°C for 4 days and 15 days whereas it increased (2-fold) in the fish exposed for 30 days. The expression of Hsp60 in the hot spring runoff fish was similar to that of the 30 days of exposed fish. Thus, Hsp60 appears to be another important player in acquired thermotolerance and adaptation.

Following 4 days of thermal exposure,* hsp90* transcript level increased by 29-fold and then decreased to 5-fold relative to control as the exposure period was extended for 30 days. Expression of* hsp90* transcripts in* Channa* collected from hot spring runoff showed similar levels of expression as that of 30 days of exposure (Figures 1(d) and 2(b)). The results of Western blot analysis corroborated with that of the transcript analysis. Seven-fold increases in the expression of Hsp90 protein was observed in 4-day exposed fishes, which eventually decreased with increase in time period. Increased synthesis of* hsp90* has been reported in fish following heat shock [22, 33, 34], besides being expressed in nonstressed conditions in different tissues and playing specialized and diversified roles [35, 36]. Thus, for Hsp90, it is difficult to assign the principal physiological role(s) that may be of increased importance at higher temperatures [22] and that may reflect the fluctuating expression pattern of its transcript in response to short and long periods of thermal exposure. The reduction in transcript level at 4 days of exposure to 36°C may indicate the effectiveness with which* Channa* is acclimated to the changing situations in terms of effects of thermal stress on protein homeostasis.

In the present investigation, the transcript level of* hsp110* increased by 43-fold relative to control when* Channa* was exposed to 36°C for 4 days. The level abruptly decreased at 15 days and equaled to or was little more than control at 30 days of exposure. The 110 kDa heat shock protein (Hsp110) has long been recognized as one of the primary heat shock proteins in mammalian cells [37]. Besides its chaperoning activity it is also a cochaperone and acts cooperatively with members of the Hsp70 family in the cytosol [38]. In the present study, the increase in the transcript level of* hsp110* at 4 days of thermal exposure happened probably to match the increase in Hsp70 content of the cell that is produced to protect the organism against the heat stress. However, while* hsp70* level decreased by a small extent by 30 days of exposure,* hsp110* transcript level abruptly decreased, probably to nullify the negative effects of its expression at high level or as its role is substituted by some other chaperone over time. However, it is interesting to note that* hsp110* transcript showed a high level of expression (~19-fold) in the hot spring runoff fish (Figures 1(e) and 2(b)), relative to control and 30 days of thermal exposure group.

As shown in Figure 1(f), the increase in expression of* hsp47* transcript decreased 0.2-fold at 4 days of exposure and increased to 1.34-fold at 15 days and then decreased when exposure period was extended to 30 days. The transcript showed similar levels of expression as 30 days of exposure in fishes living in hot spring (Figures 1(f) and 2(c)). In rainbow trout (*Oncorhynchus mykiss*) gonadal cells [39] and zebrafish embryos [40], the mRNA accumulation levels of* hsp47* have been shown to be significantly increased after heat shock. Our findings are similar to the observations made by Murtha and Keller [41] who found no significant increase in the* hsp47* expression in liver or muscle tissues of zebrafish following heat stress. However, these workers reported very high level of expression (~100%) in brain suggesting differential expression of Hsp47 in different tissues. It would be interesting to see the Hsp47 expression in brain tissue of* Channa*.

In addition to stress-inducible expression,* hsp27* is constitutively expressed in many cell types though its expression in liver tissue is low [12]. In the present study, compared to control, the expression of* hsp27* transcripts was low at 4 days and 15 days of thermal exposure, while at 30 days the expression increased to a small extent; however, the level was less than the control in all cases (Figure 1(g)). Expression of* hsp27* transcripts showed similar levels of expression as that of 30 days of exposure in samples collected from hot spring runoff (Figure 1(g)). In rainbow trout, exposed to a continuous heat shock, the mRNA level of HSPb1 increased [42]. In a study by Logan and Somero [32],* hsp27* was significantly up regulated in the most extreme time points in the 19 and 28°C acclimation groups of eurythermal fish* Gillichthys mirabilis.*

Hsp27 is known to play a role in translation inhibition during heat shock [39]. Under extreme heat stress, ectotherms have been shown to inhibit protein synthesis, possibly to limit accumulation of unfolded proteins that might damage cells [3]. The extent to which Hsp27 may be involved in translational arrest in this study is not known.

Taken together, the present findings suggest that Hsps are good candidates as ecologically relevant biomolecules used by animals to ameliorate thermal stress and play important roles in thermal adaptation. While, Hsp90 and Hsp110, besides Hsp70, are required for immediate survival of fish at high temperature, Hsp60, Hsp70 and Hsp78 are needed for long term survival at high temperature in* Channa*. We found the transcript levels of the* hsps* in fish exposed to 36°C comparable to that living in hot spring runoff, which is indicative of the hypothesis that the fish living in the hot spring runoff continuously express Hsps at levels that can be approximated by long term induction of Hsps in farmed fish raised at elevated water temperature, from 25°C to 36°C, which could possibly be a situation in severe forms of climate change and global warming. The peculiar behavior of* hsp110*, the sharp increase in expression (43-fold) at 4 days of exposure, and returning to basal level by 30 days and then remaining at a higher level (19-fold) in the perennially heat-stressed hot spring runoff fishes as compared to control or 30 days of exposed fish appear quite interesting and merit further investigation. Additional studies on liver proteome changes in* Channa* can be investigated to look for global protein expression signatures, under the same thermal climes, to fish out the proteins which might be in cross talk with the Hsps. It would also be interesting to know what happens when the hot spring runoff fishes are allowed to acclimatize to temperature of aquaculture ponds (25–27°C), asking if their* hsp* gene expression returns to control level or if their* hsp* expression level remain high. Further, the hot spring runoff fishes can be heat shocked at 41/42°C to see the heat shock response.

## Figures and Tables

**Table 1:** Primer sets used for semiquantitative RT-PCR and quantitative RT-PCR analysis of *hsp* genes in *Channa striatus*.

Primer name	Primer sequence (5′-3′)	Annealing temperature	Amplicon size (bp)
Semiquantitative RT-PCR			
*hsp70 *	F: 5′-GCATGGTGAACCACTTTGTG-3′	53°C	368
	R: 5′-CTCTGCCGTTGAAGAAATCC-3′		
*hsp78 *	F: 5′-TCCAGGCTACCCTGATTTTG-3′	60°C	360
	R: 5′-CACCATGCCTACATCCTCCT-3′		
*hsp60 *	F: 5′-C(C/T)G TCA CCATGG G(A/G/T)C CAA AGG-3′	65°C	648
	R: 5′-(C/G/T)GC CTC TCC ATC CAC ATC C/T)TC(A/C)GC-3′		
*hsp90 *	F: 5′-GGAAATCTTCCTCCGAGAGC-3′	51°C	244
	R: 5′-CCGAATTGACCGATCATAGA-3′		
*hsp110 *	F: 5′-GCAGATCTCCGCTATGCTGT-3′	56°C	242
	R: 5′-CGAATGACCCAAGTCCACGA-3′		
*hsp47 *	F: 5′-CACTGGGATGAGAAGTTCCA-3′	59°C	408
	R: 5′-AAGGAAAATGAAGGGATGGTC-3′		
*hsp27 *	F: 5′-CTT TGG GAT GCC ACA CTT CT-3′	50°C	233
	R: 5′-ATC TGG CGT CTG CTT GAT CT-3′		

Quantitative RT-PCR			
*hsp70 *	F: 5′-GTC GTG GATCTGTCCCTTGT-3′	57°C	98
	R: 5′-CTCGCTTTGAGGAGCTGTG-3′		
*hsp78 *	F: 5′-GTC TTG GTG GGT GGC TCT AC-3′	57°C	94
	R: 5′-TCA GGG TTG ATG CCC CTA GA-3′		
*hsp60 *	F: 5′-TCG CAC TGT CAT CAT TGA GC-3′	61°C	91
	R: 5′-CTT GTC TTT CAG GTC GAT GG-3′		
*hsp90 *	F: 5′-CTG GGG TCT GTC AAG CTT TC-3′	60°C	122
	R: 5′-AGG CCT TGG ACA AAA TCA GA-3′		
*hsp110 *	F: 5′-AAG CTG GAG GGC TCC TAT CA-3′	53°C	90
	R: 5′-ATA CAG CCA GTT CTC CGT GC-3′		
*hsp47 *	F: 5′-CTG ACA GAG GCT GTG GAC AA-3′	52°C	60
	R: 5′-GTC CTT CTT CCC GGA GAT GT-3′		
*hsp27 *	F: 5′-TGG GAT GCC ACA CTT CTC CG-3′	59°C	70
	R: 5′-GAT GTA TCC AGG CCA GTG CG-3′		

F, forward; R, reverse.

**Table 2:** Accession numbers of partial DNA sequences of *Channa striatus hsp* genes submitted to GenBank.

*hsps* investigated	Amplicon size	GenBank accession number
*hsp27 *	**233 bp**	KC800797
*hsp47 *	**408 bp**	KC847092
*hsp60 *	**648 bp**	KC599206
*hsp70 *	**368 bp**	JQ945974
*hsp78 *	**360 bp**	KC800798
*hsp90 *	**244 bp**	KC599208
*hsp110 *	**242 bp**	KC915027

## References

[1] Intergovernmental Panel on Climate Change (IPCC) (2007). *Fourth Assessment Report—Climate Change 2007: Synthesis Report*.

[2] Mohanty S, Mohanty BP (2009). Global Climate change: a cause of concern. *National Academy Science Letters*.

[3] Feder ME, Hofmann GE (1999). Heat-shock proteins, molecular chaperones, and the stress response: evolutionary and ecological physiology. *Annual Review of Physiology*.

[4] Roberts RJ, Agius C, Saliba C, Bossier P, Sung YY (2010). Heat shock proteins (chaperones) in fish and shellfish and their potential role in relation to fish health: a review. *Journal of Fish Diseases*.

[5] Kikuchi K, Yamashita M, Watabe S, Aida K (1995). The warm temperature acclimation-related 65-kDa protein, Wap65, in goldfish and its gene expression. *Journal of Biological Chemistry*.

[6] Mahala SC, Singh P, Das M, Acharya S (2012). Genesis of thermal springs of Odisha, India. *International Journal of Earth Sciences and Engineering*.

[7] Mohanty S, Mahanty A, Yadav RP, Purohit GK, Mohanty BN, Mohanty BP (2014). The Atri hot spring in Odisha—a natural ecosystem for global warming research. *International Journal of Geology, Earth and Environmental Sciences*.

[8] Bouchama A, Knochel JP (2002). Medical progress: heat stroke. *The New England Journal of Medicine*.

[9] Bouchama A, Dehbi M, Chaves-Carballo E (2007). Cooling and hemodynamic management in heatstroke: practical recommendations. *Critical Care*.

[10] Kakkar V, Meister-Broekema M, Minoia M, Carra S, Kampinga HH (2014). Barcoding heat shock proteins to human diseases: looking beyond the heat shock response. *Disease Models and Mechanisms*.

[11] Healy TM, Tymchuk WE, Osborne EJ, Schulte PM (2010). Heat shock response of killifish (*Fundulus heteroclitus*): candidate gene and heterologous microarray approaches. *Physiological Genomics*.

[12] Wang J, Wei Y, Li X, Cao H, Xu M, Dai J (2007). The identification of heat shock protein genes in goldfish (*Carassius auratus*) and their expression in a complex environment in Gaobeidian Lake, Beijing, China. *Comparative Biochemistry and Physiology C: Toxicology and Pharmacology*.

[13] Altschul SF, Gish W, Miller W, Myers EW, Lipman DJ (1990). Basic local alignment search tool. *Journal of Molecular Biology*.

[14] Bustin SA, Benes V, Garson JA (2009). The MIQE guidelines: minimum information for publication of quantitative real-time PCR experiments. *Clinical Chemistry*.

[15] Mohanty BP, Bhattacharjee S, Mondai K, Das MK (2010). HSP70 expression profiles in white muscles of riverine catfish *Rita rita* show promise as biomarker for pollution monitoring in tropical rivers. *National Academy Science Letters*.

[16] Bradford MM (1976). A rapid and sensitive method for the quantitation of microgram quantities of protein utilizing the principle of protein dye binding. *Analytical Biochemistry*.

[17] Laemmli UK (1970). Cleavage of structural proteins during the assembly of the head of bacteriophage T4. *Nature*.

[18] Towbin H, Staehelin T, Gordon J (1979). Electrophoretic transfer of proteins from polyacrylamide gels to nitrocellulose sheets: procedure and some applications. *Proceedings of the National Academy of Sciences of the United States of America*.

[19] Basu N, Todgham AE, Ackerman PA (2002). Heat shock protein genes and their functional significance in fish. *Gene*.

[20] Kayhan FE, Duman BS (2010). Heat shock protein genes in fish. *Turkish Journal of Fisheries and Aquatic Sciences*.

[21] Kültz D (2005). Molecular and evolutionary basis of the cellular stress response. *Annual Review of Physiology*.

[22] Logan CA, Somero GN (2010). Transcriptional responses to thermal acclimation in the eurythermal fish *Gillichthys mirabilis* (Cooper 1864). *American Journal of Physiology: Regulatory Integrative and Comparative Physiology*.

[23] Deane EE, Woo NYS (2005). Cloning and characterization of the hsp70 multigene family from silver sea bream: modulated gene expression between warm and cold temperature acclimation. *Biochemical and Biophysical Research Communications*.

[24] Lejeusne C, Pérez T, Sarrazin V, Chevaldonné P (2006). Baseline expression of heat-shock proteins (HSPs) of a “thermotolerant” Mediterranean marine species largely influenced by natural temperature fluctuations. *Canadian Journal of Fisheries and Aquatic Sciences*.

[25] Lund SG, Ruberté MR, Hofmann GE (2006). Turning up the heat: the effects of thermal acclimation on the kinetics of hsp70 gene expression in the eurythermal goby, *Gillichthys mirabilis*. *Comparative Biochemistry and Physiology*.

[26] Colson-Proch C, Morales A, Hervant F, Konecny L, Moulin C, Douady CJ (2010). First cellular approach of the effects of global warming on groundwater organisms: a study of the HSP70 gene expression. *Cell Stress and Chaperones*.

[27] Clark MS, Fraser KPP, Peck LS (2008). Antarctic marine molluscs do have an HSP70 heat shock response. *Cell Stress and Chaperones*.

[28] Leidhold C, Janowsky BV, Becker D, Bender T, Voos W (2006). Structure and function of Hsp78, the mitochondrial ClpB homolog. *Journal of Structural Biology*.

[29] Werner I, Smith TB, Feliciano J, Johnson ML (2005). Heat shock proteins in juvenile steelhead reflect thermal conditions in the Navarro River watershed, California. *Transactions of the American Fisheries Society*.

[30] Werner I, Linares-Casenave J, van Eenennaam JP, Doroshov SI (2007). The effect of temperature stress on development and heat-shock protein expression in larval green sturgeon (*Acipenser mirostris*). *Environmental Biology of Fishes*.

[31] Lockwood BL, Sanders JG, Somero GN (2010). Transcriptomic responses to heat stress in invasive and native blue mussels (genus *Mytilus*): molecular correlates of invasive success. *Journal of Experimental Biology*.

[32] Logan CA, Somero GN (2011). Effects of thermal acclimation on transcriptional responses to acute heat stress in the eurythermal fish *Gillichthys mirabilis* (Cooper). *American Journal of Physiology: Regulatory Integrative and Comparative Physiology*.

[33] Hermesz E, Ábrahám M, Nemcsók J (2001). Identification of two hsp90 genes in carp. *Comparative Biochemistry and Physiology C: Toxicology and Pharmacology*.

[34] Hooven TA, Yamamoto Y, Jeffery WR (2004). Blind cavefish and heat shock protein chaperones: a novel role for hsp90*α* in lens apoptosis. *International Journal of Developmental Biology*.

[35] Vanden Berghe T, Kalai M, van Loo G, Declercq W, Vandenabeele P (2003). Disruption of HSP90 function reverts tumor necrosis factor-induced necrosis to apoptosis. *Journal of Biological Chemistry*.

[36] Long TW, Ka HC (2008). Characterization of heat shock protein 90 in the shrimp Metapenaeus ensis: evidence for its role in the regulation of vitellogenin synthesis. *Molecular Reproduction and Development*.

[37] Oh HJ, Chen X, Subjeck JR (1997). hsp110 protects heat-denatured proteins and confers cellular thermoresistance. *Journal of Biological Chemistry*.

[38] Polier S, Dragovic Z, Hartl FU, Bracher A (2008). Structural basis for the cooperation of Hsp70 and Hsp110 chaperones in protein folding. *Cell*.

[39] Ojima N, Yamashita M, Watabe S (2005). Quantitative mRNA expression profiling of heat-shock protein families in rainbow trout cells. *Biochemical and Biophysical Research Communications*.

[40] Pearson DS (1996). Cloning and characterization of a cDNA encoding the collagen-binding stress protein hsp47 in zebrafish. *DNA and Cell Biology*.

[41] Murtha JM, Keller ET (2003). Characterization of the heat shock response in mature zebrafish (*Danio rerio*). *Experimental Gerontology*.

[42] Ojima N (2007). Rainbow trout hspb1 (hsp27): identification of two mRNA splice variants that show predominant expression in muscle tissues. *Comparative Biochemistry and Physiology B: Biochemistry and Molecular Biology*.

